# Photovoice and Health Perception in a Group of Early-Career Nurses

**DOI:** 10.3390/nursrep14030163

**Published:** 2024-08-29

**Authors:** Jakub Lickiewicz, Bettina Kolb, Jadwiga Piątek, Laura S. Lorenz

**Affiliations:** 1 Department of Health Psychology, Jagiellonian University Medical College, 31-501 Krakow, Poland; jadwiga.piatek@uj.edu.pl; 2 Department of Sociology, University of Vienna, 1090 Vienna, Austria; bettina.kolb@univie.ac.at; 3 Heller School for Social Policy and Management, Brandeis University, Waltham, MA 02453, USA; llorenz@brandeis.edu

**Keywords:** experiential learning, critical thinking, health psychology, nursing education, participatory photography, photovoice for clinical education, theory of planned behaviour

## Abstract

**Background.** Nurses’ perceptions of health are essential to decision making and communicating with clients. However, little is known about their own perceptions of this phenomenon. This study focuses on health-related beliefs among young nurses enrolled in a master’s-level nursing program using a modified photovoice methodology. **Methods.** The study population was 87 nurses undergoing a master’s degree in nursing at Jagiellonian University Medical College in Krakow, Poland, participating in an obligatory health psychology course. For the modified photovoice activity, the participants took three photos related to their perceptions of (1) health, (2) health protective factors, and (3) health risk factors. The data interpretation involved a thematic analysis of these photos and captions; a narrative analysis to distinguish between documentary and symbolic photos; and a descriptive analysis of the photo production. **Results.** Eighty-seven students completed the photography assignment. The mean age was 22.1 years (SD = 1.1). Most photos (91%) documented real-life health behaviours. Some photos (9%) used everyday objects such as sunflowers to create symbols related to health. A photo series showed a model of the human brain in different environments and activities. **Conclusions.** Student participation in the photovoice activity appeared to strengthen observation and interpretation skills, which are essential to client care. Students used this opportunity to reflect on their own lives and environments and show their perceptions of health, health protective factors, and health risks. The activity planted seeds for changes in students’ health perceptions and critical thinking. Future research could explore whether participation in a modified photovoice activity as experiential learning in a required health psychology course contributes to changes in master’s-level nursing students’ personal health behaviours and client care.

## 1. Introduction

The discipline of health psychology applies psychological methods to understand human behaviour in the context of health and illness [1]. An understanding of health psychology is vital, as nurses promote and maintain the health of their clients, prevent and treat illness, identify and diagnose factors contributing to clients’ health, illness, and dysfunction, contribute to the healthcare system, and inform health policy [2]. Understanding the linkages in clients among emotions, behaviours, and consequences and learning about theories related to motivating behaviour changes (i.e., from smoking tobacco to quitting) are foundational topics in health psychology study for master’s-level nursing students. However, little is known regarding the effectiveness of incorporating a photovoice activity into health psychology training for master’s-level nursing students.

**Health-related behaviour.** According to Ajzen’s Theory of Planned Behaviour (TPB), decisions about health-related behaviour are guided by beliefs about (a) the likely consequences of personal behaviour, (b) the perceptions of other important people about what the individual should do, and (c) the client’s ability to carry out the health-related behaviour. These three beliefs provide the basis for an individual to form an intention to engage in the behaviour. Its actual performance, however, depends on behavioural control [3]. This study and paper are focused on the health-related beliefs among early-career nurses enrolled in a master’s-level nursing program and its required health psychology course.

To support their clients, it is important for nurses (and other medical personnel) to understand the mechanisms underlying their clients’ health-related decisions [4,5,6]. At the same time, nurses’ perceptions of health are essential to their work with clients (communicating and making decisions with them). From a clinical perspective, nurses are expected to be role models of health-promoting lifestyle behaviours for their clients. Their ability to play this role effectively can directly or indirectly affect their clients’ beliefs or attitudes toward health promotion [7]. Their ability to play this role effectively also affects clients’ abilities to change unhealthy behaviours such as having a sedentary lifestyle, consuming alcohol, or smoking tobacco.

Both inside and outside the clinical setting, nurses make decisions about health-related behaviours for themselves every day. Their personal health behaviours and their related consequences affect their perceptions of and attitudes towards health in turn [8]. Factors known to impact health and physical activity among nursing students include gender, health concerns, and time per week spent searching online for health-related information [9]. Factors that predict health-promoting lifestyle behaviours in a group of nurses in a hospital setting have been shown to include hospital level (e.g., tertiary), number of years working as a nurse, nightshift status, and monthly personal income [7].

**Social perception and subjective reality.** Social perception, a dynamic and creative process, studies how reality or ideas influence human thinking, behaviour, and feelings [10]. The purpose of social perception is to create a subjective image of reality, which is affected by experiences, attitudes, and the individual’s current emotional state. Social perception is an active and creative process; an individual registers, selects, processes, and interprets received impressions and information and compares them with their current state of knowledge about the environment or topic, i.e., health [11].

Social perception enables a person to acquire and deepen their knowledge about reality and use this knowledge as the foundation for thinking about all aspects of the topic at hand. Essentially, the social perception process is a construct that involves how a person interprets and understands the social environment, creates various judgments and patterns, and classifies events, actions, and people, as well as their behaviours, including those related to health [12]. A social perception lens illuminates the patterns by which a person organises their existing knowledge, thus helping to make the surrounding world clear and understandable and significantly impacting their perception, interpretation, and assessment of reality [13].

Social perception, a complex process, consists of many elements. Modification of any aspect of perception, even minimally, may be associated with great variation in the effect of the perception process. The required health psychology course is intended to encourage accurate perceptions among nursing students of social activities such as health. The instructor added an experiential (hands-on) learning activity to encourage observation and critical thinking about health and related factors. The activity involved a structured observation and writing process intended to support the students’ ability to formulate conclusions while avoiding errors of perception. Inevitably, people perceive topics such as health in a biased way, due to the influence of our experiences and needs [14]. The hands-on learning activity used photography, discussions, and caption writing to help the students to avoid stereotypes, prejudice, and discrimination related to members of specific client groups (i.e., people who abuse alcohol) [15].

An ability to reflect on and think critically about health and maintain a balanced view that avoids stereotypes and prejudices is critical to providing quality care for clients. For master’s-level nursing students completing a required health psychology course at Jagiellonian University Medical College in Krakow, Poland, the faculty sought to incorporate an experiential learning activity that would encourage reflection, observation, interpretation, and self-awareness [16]. The chosen method was photovoice, modified into a single photo-taking assignment and class discussion intended to offer practice in the skills of observation and interpretation, similar to Dongre [17] working with medical students.

**Photovoice.** The photovoice method bridges the knowledge gap between individuals with lived experience and the people—clinicians, program administrators, and policymakers —who make decisions about their lives [18]. With photovoice, participants are visual ethnographers as they take photos to answer the questions posed, discuss their photos with each other, write captions for selected photos, and present findings (photos and captions grouped in themes) to decision makers [19]. The photovoice method is anchored in feminist, social justice, and community empowerment principles, where the combination of photography, critical dialogue, and experiential knowledge creates opportunities for marginalized groups to highlight social problems and drive social change [20].

Photovoice and other participatory visual ethnography methods have also been used with front line workers such as nurses and custodians [21,22,23]. With patients, nurses are in a position of power; however, in the clinical setting, they are in positions of less power vis à vis doctors and medical residents [22]. For the current study, the focus was the potential influence of participating in a modified photovoice study on the ability of early-career nurses studying health psychology to observe, interpret, and build their perception skills. Arguably, early-career nurses are not a marginalized group, yet they are at risk of failing to grasp the patterns and links between personal behaviour and environmental context (i.e., social perception and critical thinking), for example, a link between drinking alcohol and its ubiquitous promotion and availability. We surmised that participating in an activity like photovoice, which is known to provide practice in observation, interpretation, and critical thinking [17,24,25], would contribute to strengthened skills among early-career nurses in master’s-level training.

Photovoice has been used as a teaching tool for a range of undergraduate and graduate students in nursing and medicine [26,27,28], health sciences [24], and allied health professions [17,29]. The method has been used in nursing education to help nurses better understand clients’ experiences and learn to empathize with them [30]. Studies in Africa, Europe, North America, and south Asia found that using photovoice in clinical education led to increases in students’ abilities to recognize their shared humanity with clients [29], express their emotions and have empathy [28], engage in their learning [27], think critically, observe, and interpret [17,24,25], and apply course concepts [29].

A research gap in studies using the photovoice method in clinical education is the lack of theories behind studies and courses using the method. Ajzen’s TPB is foundational to the required health psychology course. We propose that (1) the active learning of a modified photovoice activity might strengthen student perceptions of the processes behind their clients’ health behaviours, including their statements and actions, and (2) learning from the experiential activity will create opportunities for self-understanding and empathy when nurses are in a position of power over their clients in the clinical setting.

The first author added a modified version of the photovoice method to the master’s-level nursing students’ required health psychology course, with the goals of engaging the students in their learning, encouraging a more profound understanding of health psychology when providing clinical care, and gaining deeper insights into their own health perceptions, attitudes, and prejudices when providing client care. The study aimed to analyse and compare issues related to taking pictures for a course assignment and assess the effectiveness of a photovoice activity adapted to clinical education for master’s-level nursing students.

## 2. Materials and Methods

The study population was 87 nursing students undergoing a master’s degree at Jagiellonian University Medical College in Krakow, Poland, participating in an obligatory health psychology course. The study took place between December 2023 and February 2024.

The course and photovoice activity were facilitated by a single faculty member to avoid the potential interference of any extraneous variables connected with the lead researcher’s expectations. The structured health psychology training consisted of 15 lecture hours and 15 experiential training hours (total hours = 30). The course acquaints participants with the psychological mechanisms of health, the relationship between stress and health, and theoretical models of stress. The study had three stages: Stage 1 involved the training and photo-taking activities; Stage 2 involved photo discussions during class time with the students who took the photos; and Stage 3 involved data analysis by the co-authors.

In Stage 1, with the modified photovoice activity adapted to the health psychology course, the participants were asked to take three photos related to their perceptions of (1) health, (2) health protective factors, and (3) health risk factors. They were informed about the ethical issues of taking photos, including not taking any pictures that showed clients’ faces and not taking any pictures without a person’s consent. The assignment’s instructions were purposefully open-ended, in order to encourage the students to choose for themselves how to understand the task and how to complete it. The students worked individually. They were allowed (but not required) to create a series or a story with their three photos. After finishing the photo-taking task, the students submitted their photos online to the university server.

In Stage 2, the participants uploaded three photos showing their subjective perception of health, health risks, and health protective factors. The students interpreted their photos together as a group during three course sessions facilitated by the course faculty. Each of the 87 students had an opportunity to show their photos to the group, listen to the discussion, and then explain why they took their photos and what they meant. The photovoice activity was not graded, in order to avoid competition among the students to take “good” photos and avoid any negative connotations among the photographs taken.

Following the photo discussion sessions, the students were informed about the possibility of having their photos included in a research study with complete anonymity. Each student could choose to delete their images from the course database with no impact on their course grade. The participants chose to remove a total of 40 photos from the research database: 10 each for perception and protective factors, and 20 for risk factors.

At the end of the course, the students were asked if they would agree to participate in the study. They were informed that their pictures would be anonymised and that their teacher would not be a part of the research to avoid the risk of bias. They were also assured that their decision would not affect their final grade. The participants provided written informed consent to participate, but even so, they were allowed to delete the pictures taken at any research stage without explanation. The research team started their work after the students received their grades to avoid the impression of pressure. Before the analysis, all individual data were removed from the material, so the research team would not know the identities of the participants.

In Stage 3, the data interpretation followed the approach used by an earlier study with nursing and medical students and included (1) a thematic analysis of the photos and their captions, a common approach to interpreting photovoice data [31]; (2) a narrative analysis to distinguish between documentary and symbolic photos and photos taken in a series [27]; and (3) an analysis of the photo production, or the process students used to obtain their photos, including the type of camera, the amount of time allowed for the photo-taking task, and image production strategies, e.g., taking an individual photo or taking photos in a series [27]. This process gave us better insight into the participants’ attitudes and perceptions of health. The data interpretation focused primarily on a descriptive analysis of the pictures, as recommended by Riessman [32], including their use to show specific topics and express emotion using symbols. The interpretation also followed the model provided by Dongre [17], which involved three steps: describe each photograph’s details, note the meaning of each photo from the student photographer’s perspective, and describe the faculty observations of each photo and its relationship to the learning activity [17], in our case, the required health psychology course.

## 3. Results

Eighty-seven nurses completed the photography assignment. Two students decided not to include their pictures in the study. Two men (2.30%) were in the group, while the rest of the group identified themselves as women (97.70%). The mean age was 22.1 years (SD = 1.1), with the ages being between 21 and 23. The study database has 227 photos, 79 (each) related to perception and protective factors and 69 related to risk factors. From a photo production standpoint, all students used cell phones to take their photos. The majority (184/81%) of the photos documented actual experiences; some photos were captured in the moment, while others used a constructed scenario, like a still life painting (29/11%). A few students took their photos in a series as they responded across the three prompts. The course photos responded appropriately to the prompts and showed the perceptions of master’s-level nursing students about health, health protective factors, and health risk factors. Half of the deleted pictures depicted risk factors.

The visual responses to the first two prompts illustrated consistent subjects and themes, e.g., activities that promote physical health, activities that promote mental health, and the importance of being out in nature and having a healthy work–life balance. In the content analysis, we discuss these topics in detail. The study photos showed the beliefs of the participating nurses about the consequences of personal behaviour. In the photo captions, the nurses explained their thoughts and feelings about what their photos represented. Narrations can contribute to a reflection process and changes in personal agency or beliefs that individuals have the ability to implement changes in their individual personal behaviour. Most photos documented real-life health behaviours observed using a camera (documentary photos). A few photos (24/9%) used everyday objects such as sunflowers to create symbols or metaphors related to health (symbolic photos). A few students took their photos in a series, showing a symbolic subject (i.e., a model of brain) in different environments and involved in different activities.

In the sections that follow, we provide a sampling of documentary and symbolic photos and photo series. A caption written by the photographer accompanies each image. The photo source (number and group) follows each photo caption and each caption excerpt.

### 3.1. Perception of Health and Related Strategies and Protective Factors

Seventy-nine photos showed general health perceptions, and seventy-nine photos showed protective factors. The responses to both prompts showed similar content: physical activities (e.g., riding a bike, hiking in mountains, or walking in nature or parks) and mental health activities (e.g., relaxation, reading a book, eating breakfast, holidays, or hugging loved ones). Several photos showed places that helped the photographer to feel happy and relaxed (e.g., cycling, hiking, or spending time near mountains and the sea). Related photo captions referred to pleasure, good fortune, and happiness. The photos showed health as a productive physical and mental health process, where people have the power to change their life habits. Through the photos, the photographers’ personal attitudes became visible. Their photos depicted both individual and collective notions about health.

**Figure 1:**
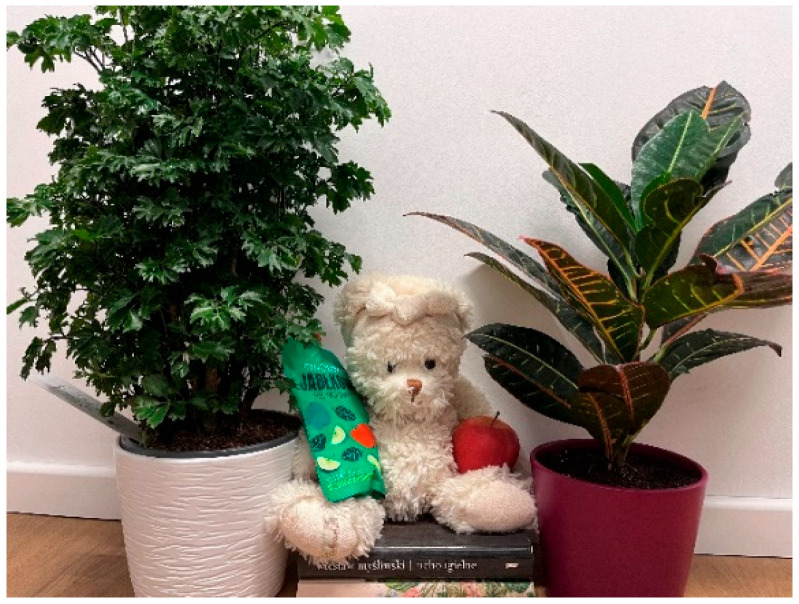
Green is Healthy (#34). **Caption:** “Green is Healthy”: plants, fruits, vegetables are beneficial to health. You need to get out of the city and into green areas as often as possible, because it also calms you down. The teddy bear in the photo is a symbol of animals, but also people and living beings. The teddy bear sits on books talking about respecting nature (Source: Master’s Nursing Student, Photo 34, Group 2).

Figure 1, titled “Green is Healthy” shows a private apartment, where an arrangement of two potted plants, healthy snacks, a stuffed toy bear, and a pile of books are placed on a wooden floor. The photographer created a still life depicting (a) actual factors contributing to health: nature, knowledge, and food, and (b) humans and animals represented symbolically by the stuffed animal. The caption describes elements that promise a healthy life and a way to control yourself and relax.

Based on the caption, Figure 1 depicts a health protective factor: spending time in nature. The photographer used house plants to depict green areas in the environment. The books suggest the importance of educating people—symbolized by the stuffed bear—about getting outside and respecting nature. The photographer appears to suggest that the health of humans is influenced by nature and the health of nature is affected by humans in turn, an example of social perception and critical thinking. The photo and caption reveal the student’s perception that health behaviour changes are possible, as conceptualized in TPB.

**Figure 2:**
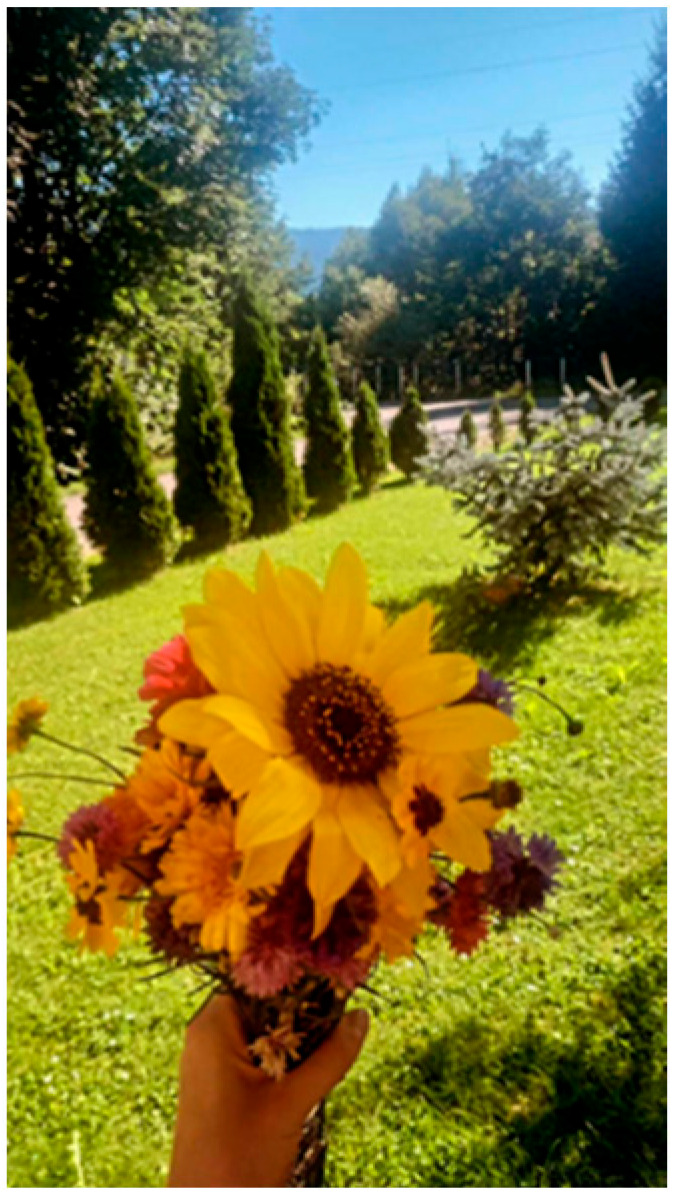
Health is Peace (#48). **Caption:** “Health is Peace”: In the foreground, a sunflower, because it is a symbol of happiness and health. The blue sky symbolizes living space. This view completely illustrates peace—which affects health (Source: Master’s Nursing Student, Photo 48, Group 4).

In Figure 2, a photo entitled “Health is Peace” shows a bouquet of flowers with a sunflower in the centre. The flower is shown in a park. A sunflower dominates the foreground and is a colourful symbol for happiness and health, in particular, mental health. In the background, the photo shows a lawn and row of trees in a park and a blue sky. The student’s caption focuses on the health behaviour of getting “into green areas as often as possible” and feeling peace while enjoying a beautiful view, which, as the photographer posits, will affect health.

This photo is reminiscent of other study photos with a similar content. Many study photos showed outside activities that participants considered as contributing to health: hiking, biking, walking the dog, and spending time outdoors. Besides the documentary aspect of walking in a park, the photographer explains in the captain that the sunflower is a symbol of happiness and health, thus referring to mental health. The phrase “this view completely illustrates peace” indicates the need for perception and critical thinking skills among the group of students participating in the study. As with Figure 1, the student appears to ascribe to the concept that behavioural change is possible.

Observation creates an opening for nurses to discuss health-related behaviours like this with their clients. A critical thinking perspective might also consider clients’ ability to reach such green areas easily and affordably, for example by walking, biking, or taking public transportation. The photographer, however, stays focused on the mental and physical benefits of being outside in nature. The photographer does not, at least in the caption, consider the contextual aspect of access to green areas, which would demonstrate critical thinking.

**Showing the way to health: strategies.** As noted earlier, notions of health and its protective factors were often intertwined in the study photos. A prime example is two photos taken by the same photographer that show a human brain in different situations: attached to a heart and sitting on a book. A photo (not shown) that depicts a brain and heart attached by a cable suggests that the mind and body (mental and physical health) are connected. As the photographer wrote in their caption:

There is a model of a heart—a symbol of the body and a model of a brain—a symbol of the psyche. They must be connected to each other for health to function. Tangled connecting cable—because staying healthy is not easy.

(Source: Master’s Nursing Student, Photo 53, Group 4)

**Figure 3:**
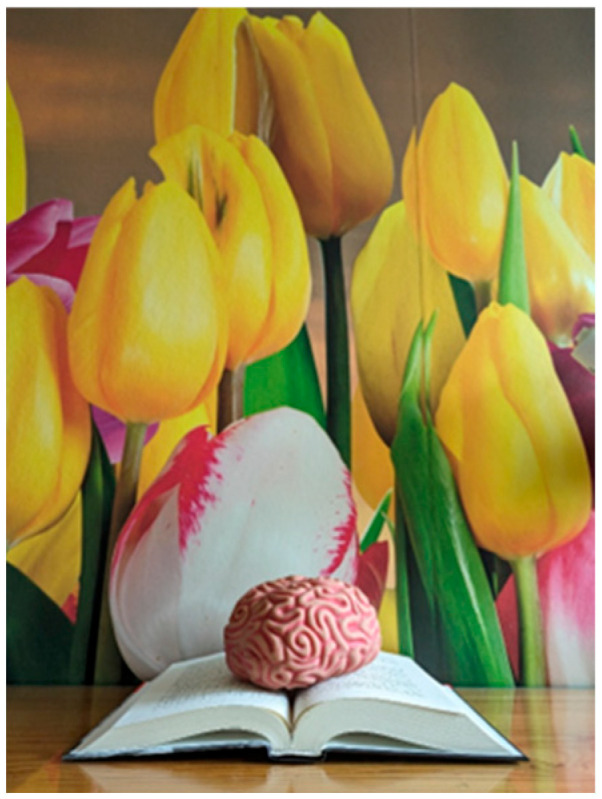
Health is Taking Care of Yourself (#61). **Caption:** “Health is Taking Care of Yourself”: Tulips—I like this photo wallpaper in my room very much because it gives hope for spring. The brain model is on a book, which means that it is learning or resting while reading. This is how you can take care of your health (Source: Master’s Nursing Student, Photo 61, Group 5).

The second photo (see Figure 3) in the series shows a model of the brain lying on a book, which represents knowledge and education. The brain and book are in a friendly environment: in front of a wallpaper showing bright yellow, white, and red tulips in bloom. In the caption, the photographer refers to the pleasant surroundings as a health protective factor. A second health protective factor shown is reading for education and relaxation, represented by the book upon which the brain is resting.

The photo and caption suggest that it is not easy to stay healthy, a potentially useful and empathic attitude when working with clients. Similar to Figure 2, this photo and caption suggest that education can contribute to healthy behaviours and good health. They appear to suggest that nurses have an educational role to play in their clients’ health, though an alternate interpretation is that clients should educate themselves.

**Protective factors.** The photos of protective factors showed a range of factors: healthy nutrition (e.g., vitamins, slow food, and vegetables), healthy activities outside (e.g., hiking, traveling, and riding a bike), social connections (e.g., friends, family members, and pets), and being in natural surroundings (e.g., streets, rivers, mountains, and parks). Most photos of protective factors were documentary photos that placed the protective factor in the centre of the photo. Examples include photos of medicine or supplements, that “keep us healthy” or protective items for hospital staff, i.e., gloves. Symbolic photos depicted strategies to achieve good mental health by addressing, for example, work–life balance. As a student wrote in the caption for her photo: “Slow Life: For physical and mental health, it is important to find time to relax and rest. The photo shows a board directing you to such a place” (Source: Master’s Nursing Student, Photo 157, Group 5).

Some photo captions presented individual notions of health using traditional Polish sayings, including “if you make your bed properly, you will sleep well”. Some played on the caption’s words; for example, a photo showing two people on a mountain peak had the caption “Happiness is the highest peak of health” (Caption, Photo 61, Group 4). Other photos revealed notions of health using phrases such as “In a Healthy Body, Healthy Mind” (Caption, Photo 62, Group 4) and “Health is Peace” (Caption, Photo 48, Group 4). Answers like these to the photo prompts do not appear to demonstrate critical thinking on the part of the students, although they have a “feel good” sense about them and indicate current-day use of traditional Polish sayings.

### 3.2. Presenting Health Risks

In total, 69 photos were taken to show health risks: only a few (3) photos were symbolic; the vast majority (66) were literal and documented risks. The photos showed three commonly accepted health risks that exist for anyone, whether client or medical staff. They included alcohol (16), fast food (5), sugar (7), and smoking (7). Common photo topics consisted of cigarettes, packaged fast food, and sweets, such as cakes or donuts. The students’ photos also documented alcohol in various types of containers (glasses, bottles, and cans) and locations (in the snow and on a shelf in the supermarket). Their locations indicated the consumption of alcohol in different social situations and locations, for example while on holiday and in the home. The photos appeared to relate to behavioural beliefs; with behavioural control, we can avoid these risk factors.

One of the student photos showed three major health risks in one image: nicotine, alcohol, and sugar. Entitled “Three Musketeers” (photo not shown), referring to an adventure novel of three noble men fighting for the French king, the image shows a bottle of lemonade, representative of sugar and fast food, a bottle of alcohol, and an ashtray with cigarettes. Sugar, fast food, and alcohol are major health risks for anyone, whether client or medical staff. As noted earlier, the photos of health risks appeared to focus on unhealthy personal behaviours, such as eating sweets, cake, cookies, or drinking alcohol in social situations, and not on contextual factors contributing to consumption. The captions represent an opportunity to engage in critical thinking about alcohol consumption, for example, that colourful packaging contributes to consumption:

Alcohol kills: Consuming excessive amounts of alcohol can lead to many harmful health effects, such as liver damage, mental disorders, cardiovascular problems, and the risk of addiction. Alcohol can also negatively affect cognitive ability and motor function, which increases the risk of accidents and injuries. In Poland, almost every store has massive shelves with alcohol, which encourages consumers to buy it through colourful labels. Referring to the title of addiction, diseases and driving under the influence of alcohol very often contribute to death so that alcohol consumption can be classified as health risk factors.

(Source: Master’s Nursing Student, Photo 89, Group 3)

**Figure 4:**
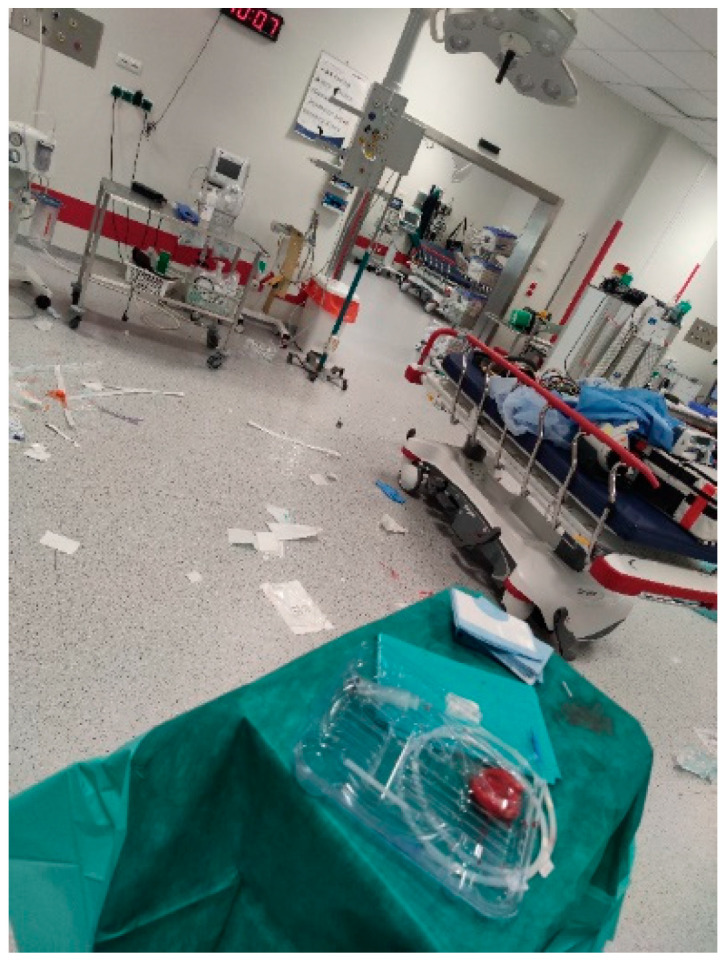
Work Life Balance (#107). **Caption:** “Work Life Balance: Emergency room. Chaos. Disorder. A remnant of the ‘acute situation’ with patient. There was a resuscitation. It takes a toll on the psyche. Cardiac arrest. It’s hard to strike a balance when it’s a difficult situation for the patient’s family and staff. It’s hard to break away from it. Risks of health disorders-stress” (Source: Master’s Nursing Student, Photo 107, Group 4).

The student photos also showed health risks from the environment, including smog, dangerous streets for pedestrians, and stressful situations in hospital work. Depicting dangerous or stressful situations in hospital work suggests that the personal health of nurses is put at risk in their work environment. Figure 4 shows a student’s photo of stress in a hospital emergency room.

Another example is a caption for a study photo (not shown): “Nurses deal with this daily. Risk factors—cannula, used needles. A nurse’s behaviour at work is important for her safety” (Source: Master’s Nursing Student, Photo 104, Group 4). Study photos and captions that focused on nurses’ work environment centred on nurses’ health and safety, not on the health and safety of clients. Both types of photos represent responses to prompts that were intentionally open-ended to encourage creativity and self-reflection, unusual activities in nursing education in Poland to date. In sum, the student photos and captions shared (1) knowledge about health, (2) health-related beliefs, (3) awareness about the relationship between physical and mental health, and (4) the positive and negative consequences of health behaviours. Some photos can be interpreted without the caption, while the majority need the caption to point the viewer towards the photo’s meaning. Health perception and critical thinking are demonstrated in photos and captions that connect mental and physical health and connect environmental factors and health-related behaviours. Reflecting on health risks in the work environment suggests critical thinking about their chosen career and its work context.

## 4. Discussion

This study aimed to describe the main issues related to taking pictures for a course assignment and assess the effectiveness of a photovoice activity adapted to clinical education for master’s-level nursing students. The study participants were master’s-level nursing students with 3 years of bachelor training and 1 to 2 years of clinical work experience. They completed the modified photovoice activity as part of a required health psychology course.

In society today, young people focus primarily on images and less on text [33]. A participatory photography approach to active learning in the course was expected to stimulate the students to think more deeply about the course concepts and their personal perceptions and attitudes towards health and health behaviours—their own and their clients’. From our perspective, how we teach is as important as what we teach—the method and the content are both essential to learning. Student participation in the photovoice activity was seen as essential to building skills such as observation, interpretation, and critical thinking, all of which are essential to client care [34]. As described above, some students’ photos and captions demonstrated these skills, while others did not represent the deeper learning sought. It is important to note, however, that a longer photovoice project would provide multiple opportunities to take photos, discuss them, and write captions, which would create greater opportunities to practice perception and critical thinking; however, a more extensive photovoice activity was not feasible for the students in this study and course.

**Increased understanding.** The photovoice method engaged the students and appeared to facilitate a deeper understanding of the topics covered during the course than could have been accomplished by imparting content without an active learning component. John et al. saw similar results [27]. In their evaluation of the activity, the students reported that the modified photovoice exercise helped them to think critically, engage in self-directed learning, and strengthen their communication. The photos depicted the reality of health in the students’ context, a classical contribution of photography to our understanding of the world around us [35].

Although the visual material did not show strategies against substance abuse, in their captions, the photographers showed critical thinking about the environment as a health protective factor (parks) and a health risk (city streets). Future research could aim to understand how nurses’ and nursing students’ perceptions of health might affect their work with clients and their personal health behaviours.

Participating in a photovoice activity appears to allow for gaining better insight into one’s personal, unconscious attitudes, for example, that consuming alcohol contributes to social activities (i.e., holidays). For some students, the activity was an opportunity to vent negative emotions, as seen in the comments (“We will die in this work”). The students appeared to agree that participating in photovoice as an experiential learning activity strengthened their conceptual understanding of TPB. As one student commented in their written evaluation of the course: “The pictures allowed me to understand them [the course concepts] better”.

**Returning to Ajzen’s theory.** Taking pictures to answer the course prompts provided an opportunity for the students to gain insight into the domains of Ajzen’s TPB: behavioural beliefs, normative beliefs, and control beliefs [36]. The photos appeared to act as a bridge connecting psychological theory and client care. The activity forced the students to reflect on their own behaviours, which may, in future, contribute to empathy and strategic support for clients facing similar risks and circumstances. Future studies might focus on the theory of change and practical skills to use with a client or themselves to encourage behaviour changes. Sharing techniques and exercises might help nurses to apply Ajzen’s Theory of Planned Behaviour in their own lives and in their work with clients.

Photovoice is more than taking photos. We suggest that the process of creating captions is as important to student learning as taking pictures. Writing the captions creates an opportunity for the participants to reflect critically on their behaviours and actions, and how the environment impacts behaviours. The captions open up the “frame” of the photo and what we can “see” in it. For example, we did not always see health strategies in the photos, but we read about them in the captions and heard about them in the photo discussions.

**Critical thinking about environmental factors.** The activity illuminated behavioural and normative beliefs, and, to a lesser extent, beliefs related to behavioural control. An increased emphasis on developing perception and critical thinking skills and understanding the role of the environment—not just personal behaviour—in health may be needed. The students were successful at focusing on their own behaviours and the common risks surrounding them (e.g., vending machines with junk food and a challenging work environment). There appeared to be less understanding related to the structural or environmental issues related to health and its protective and risk factors. A greater focus on the social determinants of health could strengthen students’ perception learning during the course and the photovoice activity. The literature suggests that photovoice might be an effective tool for teaching medical personnel about the social determinants of health [37], as well as serving as an educational tool for nursing students. An increased understanding of the social determinants of health could potentially, in turn, reduce stigma and prejudice related to unhealthy behaviours and contribute to identifying environmental risks and ways to avoid them or to reduce their impact on personal and community health.

Another theoretical framework that might contribute to perception and critical thinking skills is the socio-ecological model, which is often used to frame photovoice findings and identify opportunities for impact at different levels: personal, relational, program/community, and societal/policy [38], though, to our knowledge, this has not been implemented in any nursing or nursing education studies using photovoice to date. We suggest that adding an exercise at the end of the photo discussion session asking the students to place their photos in a domain of the socio-ecological model and discuss their placements could deepen these students’ understanding of the broader environmental factors influencing health behaviours and strengthen their perception and critical thinking skills.

**Stress in the work environment.** Working in a hospital is impacted by the individual attitudes of nurses or other hospital staff and by the organisational and structural conditions in a hospital workplace that contribute to a healthy lifestyle in the workplace, such as the duty rotation, canteen, training opportunities, and meeting and recreation rooms for the staff. In addition, it is important to create opportunities for students to reflect on the wider environment’s social and physical characteristics that contribute to health or lack of it. For example, a photovoice study about diabetes management in an underprivileged neighbourhood in a southern Italian city showed that, while the general societal context supports blood sugar monitoring, which is impacted by personal traits and attitudes, there is less awareness about the social and physical characteristics of the everyday life context that influences diabetes control [39]. Similarly, research on the work environment could illuminate the factors contributing to stress and burnout for nurses and suggest new strategies to address the structural issues influencing nurses’ health.

A potential benefit for nurses and nursing students participating in a photovoice activity is stress relief. Stress and burnout are important issues in nurses’ work [40]. The literature suggests strategies like mindfulness and meditation to deal with work-related stress [41]. The participatory photography aspect of photovoice provides an opportunity to express emotions and thoughts more easily than might be possible when speaking alone. Photovoice can be a fun and stimulating activity for any participant. Future research could also explore whether taking and discussing pictures might help to diffuse nurses’ negative emotions and deal with stress in the workplace. There are some hints of this already, as a previous study mentioned the role of therapeutic photography in a group of nurses [42].

Can a photovoice activity focused on health and its protective and risk factors influence participants’ health-related behaviours? Our study was not intended to answer this question. The early-career nurses’ photographs and captions indicated that they knew that some types of food are unhealthy, that smoking is bad for health, and that physical activity promotes both physical and mental health. They pointed out the connection between mental and physical health, and the relationship between human health and the health of the natural world. Will an increased understanding of their own health behaviours, explored using photovoice, help them to help their clients or advocate for changes to the environment that contribute to protective factors? Advocacy is a common purpose of projects using the photovoice method [43], and this course-focused photovoice activity did not include an advocacy purpose or component. Incorporating an advocacy component into the course may or may not be acceptable for the master’s-level nursing program. Exploring advocacy possibilities in the course and answering the question posed above will require further discussion and research.

**Reflecting on the participants and their experiences with the activity.** As mentioned before, the study participants were young people, with most of them in their early 20s, meaning that they might perceive themselves as invulnerable and able to deal with unhealthy behaviours like a lack of sleep or the overuse of stimulants. In future, it might be beneficial to study the same factors in a group of more experienced nurses to understand their perceptions of health factors in their lives and work and to explore whether participating in a photovoice activity would contribute to improved health behaviours for them personally.

In their course feedback, the participants expressed satisfaction with the assignment and enjoyment in the task of taking photos, writing captions, and discussing them with others. They used this opportunity to reflect on their own lives, health, health protective factors, and health risks. The photos were a visual anchor for their thoughts and feelings. Most photos and captions focused on the personal experiences of the photographers. Only a few captions focused on clients. We cannot know whether empathy with oneself and one’s peers could translate in future to empathy with clients.

During the class discussions, the photos allowed for sharing ideas on health strategies and behaviours, as well as health perceptions. Throughout the course, the content focused on learning how to work with clients on their health behaviours. The modified photovoice activity provided an opportunity to reflect on their personal health, attitudes, and behaviours, and was a safe way for the students to reveal something about themselves. The assurance of confidentiality in the classroom provided them with an opportunity to feel safe sharing their knowledge about negative health behaviours like drinking and smoking.

The photovoice activity created an opportunity to exercise free will, agency, and power. The participants were free to decide what and how much they wanted to show and say about themselves and their health behaviours. In psychology, this revelation process is called “disclosure” [44]. Photovoice appears to be an opportunity to provide true and revealing answers as compared to completing a questionnaire. Pictures become another way to observe, document, and analyse personal health attitudes and norms.

In sum, incorporating an adapted photovoice activity into a required health psychology course for master’s-level nursing students appeared to engage students in their learning and lead to insights on the personal and environmental factors contributing to health and related protective and risk factors for some. The students successfully focused on their own behaviours and on the common or normative risks surrounding them. Their photographic and written responses to the questions illustrated common thinking patterns, e.g., that getting outside contributes to physical and mental health and drinking alcohol is a health risk factor. They appeared to exhibit a lower level of understanding related to structural or environmental health issues that would indicate perception and critical thinking. A greater focus on the social determinants of health or other theoretical frameworks could strengthen students’ learning during the course and the photovoice activity. Incorporating an additional experiential learning activity into the course, such as grouping the photos into themes or a theoretical framework’s domains, could strengthen nurses’ application of Ajzen’s TPB in their own lives and in their work with clients.

**Limitations and strengths.** The study has some limitations. All participant data were self-reported and subjective. In addition, the study took place in a specific context, and we cannot generalize the findings to all health psychology courses for master’s-level nursing students. Also, the modified photovoice activity was brief and did not allow for multiple opportunities to take photos, discuss them, write captions, or group them into the domains of a theoretical framework, all of which arguably can contribute to deeper critical thinking about the topic at hand [45]. As noted by Duijs et al. [43] (p. 1) “critical consciousness emerged from an iterative process”. Thus, a brief photovoice activity can plant seeds for critical thinking, i.e., consciousness-raising, but cannot be expected to lead to dramatic changes in perception, critical thinking skills, or critical consciousness among students. In turn, a brief, course-based qualitative study such as this is not intended to “measure” whether participation in a modified photovoice activity affects changes in participants’ health-related behaviours, attitudes, or beliefs, or changes in their future health behaviour work with clients. We gain a glimpse, however, of their health-related perceptions through their photographs and captions. Finally, we suggest that the open-ended nature of the modified photovoice assignment is a strength, as it appeared to allow for student creativity and contribute to a sense of agency among the participants.

## 5. Conclusions

Student participation in the photovoice activity created an opportunity to build observation and interpretation skills, which are essential to client care. The students’ photos and captions demonstrated their perceptions of health, health protective factors, and health risk factors and appeared to support the view that health behaviour change is possible. Some photos and captions demonstrated the students’ perception and critical thinking skills, while others did not appear to represent the deeper learning sought. We suggest that photovoice is more than taking pictures. The process of writing captions created an opportunity for the students to reflect critically on their behaviours and actions and how the environment impacts health behaviours. In their captions, multiple students showed the environment as both a health protective factor (parks) and a risk factor (city streets). The modified photovoice activity appeared to provide an opportunity for the students to reflect on their personal health, attitudes, and behaviours and reveal something about themselves in a safe way. Taking photos and writing about them provided an experiential opportunity for the students to observe, document, and analyse their health attitudes and norms. Further research is needed to understand whether participating in a brief photovoice activity on a required health psychology course can contribute to behavioural changes among the participating master’s-level nursing students in their own lives and their work with clients. Incorporating an activity for students to identify themes in their photos or group them into the domains of a theoretical framework could provide an opportunity to strengthen the impact on the perception and critical thinking imparted by participating in a modified photovoice activity on a master’s-level health psychology course for early-career nurses.

## Data Availability

The data might be made available after direct contact with the corresponding author.

## Public Involvement Statement

The participants were not involved in the research.

## Guidelines and Standards Statement

This manuscript used the Standards for Reporting Qualitative Research (SRQR) for qualitative research.
